# Prenatal mercury exposure and offspring behaviour in childhood and adolescence

**DOI:** 10.1016/j.neuro.2016.09.003

**Published:** 2016-12

**Authors:** Jean Golding, Steven Gregory, Alan Emond, Yasmin Iles-Caven, Joseph Hibbeln, Caroline M. Taylor

**Affiliations:** a Centre for Child and Adolescent Health, University of Bristol, UK; b National Institute on Alcohol Abuse and Alcoholism, National Institutes of Health, USA

**Keywords:** ALSPAC, Prenatal mercury exposure, Dietary fish, Child behaviour, Adolescent behaviour

## Abstract

**Background:**

There is considerable discussion over the possible harm caused by fetal exposure to mercury, but evidence of such harm is contradictory at levels commonly found in populations with moderate intakes of fish. Further information is needed to inform debate and clarify policy recommendations.

**Material:**

Data were collected prospectively for the Avon Longitudinal Study of Parents and Children (ALSPAC). Whole blood taken in the first half of pregnancy was assayed for mercury. The outcomes were offspring behavioural assessments collected using the Strengths and Difficulties Questionnaire at seven time points between ages 4 and 16–17 years; five were completed by the mother and two by the teacher. Socioeconomic and biological confounders were first taken into account; further analyses added maternal blood selenium. Separate analyses compared the relationships between prenatal mercury levels and behaviour traits treated as continuous measures in women who ate fish with those who ate no fish in order to determine whether the relationships differed; the hypothesis was that fish consumption had benefits on the brain and masked any mercury effects. In order to prevent Type II errors, the P value for significance was set at 0.10.

**Results:**

Prenatal mercury measurements and offspring behaviour results were available for between 2776 (at 47 months) to 1599 mother-child pairs (at 16–17 years). Even given a P value of 0.10, the number of significant results was no greater than expected apart from the relationships with peer problems at 4, 6 and 10–11 years where the relationships with prenatal mercury were negative (i.e. the greater the level of mercury the fewer the problems the child had with his/her peers). There were no significant differences between the associations with mercury found among the offspring of women who ate fish in pregnancy and those who did not, nor did adjustment for selenium make a difference.

**Conclusions:**

There were no adverse effects of maternal prenatal mercury levels on the behaviour of the offspring. A similar lack of relationship was found when the analyses were confined to those offspring whose mothers had eaten fish in pregnancy, and no consistent differences were found between the fish and non-fish eaters.

## Highlights

• There is controversy as to whether prenatal mercury levels are associated with adverse child behaviour, especially if the mother does not eat fish.
• This study compares the relationship between maternal total blood mercury in the first half of pregnancy and child behaviour at seven time points.
• No evidence was found to suggest that the mercury level was associated with adverse behaviour, whether or not the mother ate fish.

## 1 Introduction

In spite of discussion over many years there is still considerable controversy as to the possible adverse effects of mercury on the developing brain, particularly in relation to its presence in sea food. This has resulted in contradictory and confusing advice to pregnant women with the result that many have reduced their consumption of fish (Oken et al., 2003) in spite of the accumulating evidence of the benefits to the child when the mother has consumed fish in pregnancy.

In 2007 we published the results of analyses of the Avon Longitudinal Study of Parents and Children (ALSPAC) which showed that, even after adjustment for multiple factors, prenatal consumption of fish was associated with no deleterious outcomes to the offspring in regard to child behaviour as reported by the mother at age 7 (Hibbeln et al., 2007). We did not consider the effects of mercury at that time as the blood samples had not been assayed at that point. We have recently had measures of total maternal blood mercury available for analysis and have shown that the early development of the child in the preschool period (measures of fine and gross motor, social and communication skills at 6, 18, 30 and 42 months) was not influenced adversely by the maternal blood mercury in the first half of pregnancy (Golding et al., 2016a). Indeed, there were positive associations even after adjustment for social and maternal lifestyle factors, indicating that the higher the total blood mercury, the more advanced the child’s development.

These findings have prompted investigation of possible effects of prenatal mercury exposures on offspring behaviour in this cohort. The literature on this association is conflicting. A study of an Inuit population in Arctic Quebec (where exposure is greater from consumption of sea mammals rather than fish), showed an increase in attention problems and disruptive behaviour at age 11 with increasing prenatal mercury exposure (Boucher et al., 2012). After adjustment a study in Massachusetts showed that maternal mercury levels in pregnancy were related to inattentive and impulsive behaviours at age 8, but that consumption of fish provided protection (Sagiv et al., 2012). Conversely in the Seychelles archipelago, where pregnant women were eating fish daily, their prenatal mercury levels were unrelated to the behaviour of their offspring at 5 years (Myers et al., 2000); at age 9 there was evidence of a negative association between prenatal mercury exposure levels and hyperactive behaviour (Myers et al., 2003); and at age 17 the higher the prenatal mercury exposure the less likely the teenager to have had problem behaviours at school and also less likely to have indulged in substance abuse (Davidson et al., 2011); at 19 years there was no association with positive or negative affect (Van Wijngaarden et al., 2013). Thus these studies suggest the hypothesis that the prenatal exposures to mercury have no adverse effects on subsequent child behaviour if the mother eats fish, but not otherwise.

In the present study, we used an unselected population of pregnancies to investigate the relationship between prenatal mercury exposure and behaviours of their offspring, to determine whether relationships differ according to whether the mother had consumed fish during pregnancy.

## 2 Material and methods

### 2.1 The study sample

The ALSPAC study aimed to enrol all pregnant women residing in Avon (a geographically defined area that includes the city of Bristol, smaller urban towns, and rural areas about 120 miles west of London, UK) with an expected delivery date between 1 April, 1991 and 31 December, 1992. The study enrolled 14,541 pregnant women, estimated as about 80% of those eligible. Its stated aims were to evaluate genetic and environmental influences on health and development, including environmental factors measured prospectively during pregnancy (Golding, 2004, Boyd et al., 2013). The study website contains details of all the data that are available through a fully searchable “data dictionary”: <http://www.bris.ac.uk/alspac/researchers/data-access/data-dictionary/http://www.bris.ac.uk/alspac/researchers/data-access/data-dictionary/>.

### 2.2 The outcome measures

The Strengths and Difficulties Questionnaire (SDQ) was developed by Robert Goodman from previous versions of behaviour scales used in the UK such as that of Rutter (1967), and its later adaption, the Revised Rutter (Elander and Rutter, 1996). It comprises 25 statements and is designed to measure prosocial, hyperactive, emotional, conduct behaviours and peer relationships, each of these scales being derived from 5 items. Each item assesses different aspects of the child’s behaviour in the last 6 months using 4 response options (‘Not true’, ‘Somewhat true’, ‘Certainly True’, ‘Don’t know’).

The SDQ is well validated (Goodman et al., 1998, Goodman and Scott, 1999) and has been shown to correlate well with other measures of psycho-pathology (Goodman, 1997). A total behaviour difficulties score is derived from summing the hyperactive, emotional, conduct and peer problems scores; for each of these scores, the higher the score the worse the behaviour. In contrast, the higher the prosocial score the more desirable the behaviour. In general, the statements referred to the past 6 months. In this study we used the scales derived from those completed by the chief carer (usually the mother) when the offspring was aged 4, 6, 11, 13 and 16 years. In addition, we used the results from the child’s primary school teacher who completed the SDQ at the end of school years 3 and 6 (ages 7–8 and 10–11 years). We have analysed each score separately on the basis that the effects of a particular exposure in pregnancy may show an effect at particular stages of development, but not at others. For each domain, apart from the total difficulties score, the range of possible scores is from 0 to 10; in order to ensure that the maximum amount of data was used, where the domain was missing data on 1–2 of the five questions, the score was prorated based on the answers to the other questions in that domain. This general adjustment was applied to <5% of scores.

### 2.3 Measurement of maternal prenatal mercury and selenium

Whole blood samples were collected in acid washed heparin vacutainers (Becton and Dickinson) by midwives as early as possible in pregnancy. Samples were obtained from women in two of the three Health Authority areas of the recruitment region. Altogether there were 4484 samples collected at gestational ages ranging from 1 to 42 weeks, median 11 weeks, mode 10 weeks; interquartile range 9–13 weeks. The social background of the women who gave such samples did not differ from the rest of the ALSPAC population apart from being slightly older and more educated (Taylor et al., 2013). Samples were stored at 4 °C at the collection site and then sent to the central Bristol laboratory within 0–4 days. These samples were kept at room temperature for up to three hours during transfer, and were stored at 4 °C as whole blood in the original tubes for 18–19 years before being sent for analysis.

The method of assay of mercury and selenium has been described in detail elsewhere (Golding et al., 2013). In brief, the laboratory of Robert Jones at the Centers for Disease Control and Prevention (CDC) developed methods to prepare the samples for analysis of whole blood mercury as well as of lead, selenium and cadmium (CDC method 3009.1). Clotted whole blood was digested to remove all clots, before being analyzed using inductively coupled plasma dynamic reaction cell mass spectrometry (ICP-DRC-MS). Two levels of bench quality control (QC) materials as well as a blind QC material were used for daily quality control.

Of the 4484 samples, the assay failed for 350 assays of mercury and 197 of selenium; All selenium measures were above the level of detection (LOD), but three of the mercury levels were below the LOD of the assay (0.24 μg/L). For these samples, in consideration of the distribution of the mercury levels, a value of 0.7 times the LOD value was considered to be a better estimate of the value than taking a mid-point. The range of mercury levels was from below the limit of detection to 12.76 with a median of 1.86 μg/L. Valid levels of selenium were available for 4287 pregnancies. The range of selenium levels was from 17.0 to 324.1 with median 108 μg/L. The correlation between levels of mercury and selenium was 0.338.

### 2.4 Maternal fish intake

Information was collected from the mothers using four questionnaires mailed to the women during pregnancy. Dietary consumption was assessed using a food frequency questionnaire (FFQ) administered at 32 weeks gestation; this asked the number of occasions per time interval that the woman currently ate specific types of food, and the most frequently consumed types of milk, fats, and bread (Rogers and Emmett, 1998). Women were offered the assistance of an interpreter or interviewer if they did not speak English or needed help to complete the questionnaire. The questions on seafood consumption (specifically, three questions concerning the frequency of consumption of white fish, oily fish, and shellfish, respectively) were obtained by asking the woman approximately how many times she ate each, with the options Never or hardly ever; About once in 2 weeks; Once, twice or three times a week; More than three times a week. The reports were partially validated by comparing responses with levels of DHA measured in maternal prenatal red blood cells, which indicated strong positive correlations (Williams et al., 2001). For the present study fish-eaters were identified as those who replied that they had either eaten oily or white fish (or both) more often than ‘never or hardly ever’.

### 2.5 Potential confounders

In this study we allowed for a variety of social factors: (a) a family adversity index (FAI) which is derived from 38 factors present in pregnancy including maternal depression and anxiety – used as a continuous scale (Bowen et al., 2005); (b) housing tenure (public housing v. rest); (c) household crowding (no. of persons in household/no. of rooms available); (d) stressful life events in first half of pregnancy (sum of 44 possible events – treated as continuous scale); (e) smoking at 18 weeks gestation (yes v. no); (f) alcohol consumption at 18 weeks (yes v. no); (g) maternal age at birth; (h) parity (no. of previous deliveries); (i) highest maternal education level achieved; (j) whether the child was breast fed and (k) sex of the child. We did not allow for birthweight or gestation as we considered these to be possibly on common pathways to the behaviour traits.

### 2.6 Statistical analyses

The statistical analyses first assessed the unadjusted associations between prenatal mercury and each of the developmental outcomes measured on continuous scales using multiple regression. The analyses were then adjusted for the possible confounders described above (Model A). This model was then repeated for children whose mothers ate fish during pregnancy, and those who did not. Finally we incorporated selenium into the analyses by adding it as a covariate (Model B) since it has been suggested that methylmercury may inhibit the functionality of selenium (Raymond et al., 2012). The aim of the analyses was to assess whether there were adverse associations between prenatal mercury levels and offspring behaviour, and whether there were differences in the associations among fish eaters compared with non-fish-eaters. Consequently interactions between these two groups was sought in the models including all children.

Since these analyses were undertaken to assess possible adverse effects of mercury exposure we were anxious to avoid Type II statistical errors, and consequently made no allowance for multiple testing, and set the level of significance at P < 0.10.

## 3 Results

### 3.1 Mercury and fish intake

**Fig. 1:**
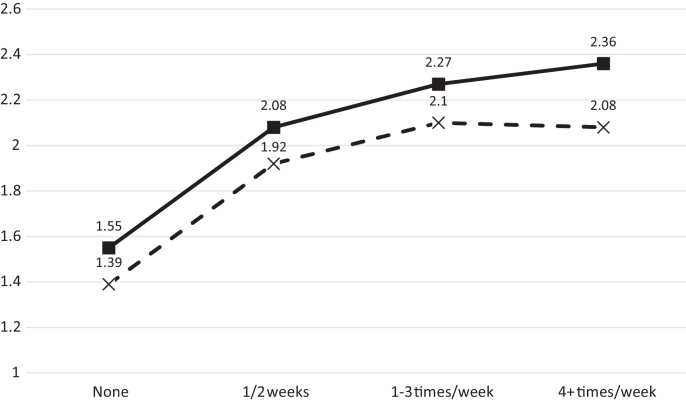
The median levels (μg/L) of maternal blood mercury according to the frequency of fish intake. The solid line denotes oily fish and the dashed line white fish.

Although it is well known that fish contain some mercury, and that oily fish have higher levels than white fish, seafood intake accounts for only a small portion of the total blood mercury (Golding et al., 2013). The way in which the median mercury level varies with the frequency of fish intake in pregnancy is demonstrated in Fig. 1, which shows that, to a certain extent, the more fish eaten, the higher the blood mercury level, but that the biggest difference is between the non-fish eaters and those that eat at least some fish. On this basis we have analysed the group of children whose mothers ate no fish with those who ate fish in looking at the effects of prenatal mercury on child and adolescent behaviour.

### 3.2 Other factors associated with blood mercury level

We show elsewhere (Golding et al., 2016b) the way in which the mercury level varied with maternal age (the older the higher the blood Hg), parity (the more previous pregnancies the lower the Hg), maternal education (the more advanced the level the higher the Hg level), prenatal smoking (associated with lower blood mercury), prenatal alcohol (increasing levels of Hg with increasing alcohol intake), and housing tenure (those in owner occupied housing had the highest, and those in public housing had the lowest mean levels of Hg). All these associations were significant at P < 0.0001.

### 3.3 Prenatal fish consumption and offspring behaviour

The unadjusted mean behaviour scores are shown for each of the seven behavioural assessments in Table 1, separately for offspring of women who ate fish during pregnancy, those who did not, and all women combined. It can be seen (by comparing the confidence limits) that there were many significant differences between the children of the fish and non-fish eaters. All the significant differences indicated that, on average, the offspring whose mothers did not eat fish in pregnancy had worse behaviour than the offspring of fish eating mothers.

### 3.4 Total behavioural difficulties and mercury

In Table 2 we compare the unadjusted and adjusted relationships between maternal prenatal blood mercury and the total behaviour difficulties score at each of the seven ages. For the unadjusted data, of the 21 analyses, 13 were associated at the 0.10 level (9 at the 0.01 level); all showed a negative association – i.e. the higher the mother’s blood mercury, the better the reported offspring behaviour. On adjustment, however, only three associations remained significant at the 0.10 level (two at the 0.01 level) – all were at age 47 months and all were negative. None of the analyses of the seven time points showed significant differences in relationships with mercury between the fish and non-fish eaters.

### 3.5 Specific behaviours

Similar analyses have been undertaken for each of the five different SDQ subscales in (Supplementary Tables 1–5). In each set of analyses we consider the unadjusted and the adjusted regression coefficients. Although we carried out two adjustments (Model A and Model B), the results for each were practically identical, and we only report the results of Model B in these tables. Those results showing P < 0.10 are summarised in Table 3.

There were 21 models for each specific behaviour, 105 overall. Thus one would expect 2 results to have P < 0.10 for each behaviour, and 10 overall. In fact overall 15 showed this level of significance, with hyperactive, conduct, emotional and prosocial behaviours having the expected numbers (2, 3, 2 and 1 respectively), and peer problems showing slightly more than expected (7).

On examining the peer problems in more detail it can be seen that there were significant unadjusted associations for all but one age group (the Teacher assessment at age 10–11). After adjustment three of the different ages showed associations at P < 0.10: at 47 and 81 months and 13 years (Table 3 and Supplementary Table 4). At each age the association was negative for both the fish and non-fish eaters and for the combined group of women, implying that the higher the mother’s blood mercury the lower the level of peer problems.

## 4 Discussion

We assessed 126 possible associations between offspring behaviour (total difficulties and the specific behaviours) and prenatal mercury exposure, and took a P value of 0.10 to ensure that no adverse (positive) effects were missed. After adjustment only 18 were significant at this level, and the majority of these indicated that the higher the prenatal mercury level the better the offspring’s behaviour (Table 2, Table 3). Thus, there was little to indicate that there were more ‘significant’ effects than expected, with the possible exception of the peer problem subgroup where 7 of 21 models had P < 0.10.

The combined scales of hyperactivity, conduct, emotional, and peer problem behaviours (the total difficulties score) only provided significant findings at one age (47 months), but this was no more than expected. Again it is noteworthy that all these relationships were negative, implying that the level of prenatal exposure to mercury was not associated with worse behaviour in the offspring. There was no indication that the relationship between prenatal mercury and behaviour differed between fish eaters and non-fish eaters apart from the difference between emotional scores (Table 3), but there were 42 tests for interaction so this was no more than would be expected by chance.

We assessed the unadjusted differences between the offspring of the fish and non-fish eating women as a background to our analysis of whether there are likely to be any differences in the relationships between prenatal mercury and offspring behaviour. This forms the basis of our study. We have shown elsewhere, using the ALSPAC population, that although seafood is not by any means the sole source of blood mercury, on average the women who eat fish do have higher levels of blood mercury than those who eat no fish (Golding et al., 2013). It has also been reported that the mercury derived from seafood is more likely to be methylmercury, and less likely to be inorganic mercury, but that both forms cross the placenta readily (Ask et al., 2002); it is assumed that methylmercury is more likely than inorganic mercury to have adverse effects on the brain of the developing fetus (Mahaffey et al., 2011). However there was no evidence that there were differences between the two groups; our results do not support those of Sagiv et al. who demonstrated a protective effect on impulsive and inattentive behaviours (equivalent to our hyperactive scale) only if the mother ate fish, since we found no difference between the behaviours of the offspring of the fish-eating mothers.

In a previous paper we reported no significant associations with adverse behaviour at age 81 months apart from that with an increased risk of poor prosocial behaviour in the children of women who did not eat fish (Hibbeln et al., 2007), but here we consider the behaviour scales as traits reported by both mother and teacher, and used the whole extent of the traits, rather than looking solely at the lower decile. We had postulated that the benefits of fish consumption might mask adverse effects of mercury, and that by analysing the women who ate no fish separately we might reveal any adverse effects. We have analysed the child’s early development in this way but shown no such difference (Golding et al., 2016a): both groups revealed beneficial relationships with increasing prenatal mercury levels.

Our results here differ from some of the few published studies of child behaviour; however most of those studies were set up because of high population levels of mercury or other pollutant exposure. This is particularly true of the women in the Seychelles who tended to eat fish every day (Davidson et al., 2011), studies among the Inuit populations where exposure again was more likely to be from sea mammals (Boucher et al., 2012), and a study in Massachusetts undertaken because of high local exposure to PCBs (Sagiv et al., 2012). Of those with information on offspring behaviour only ALSPAC can be presumed to be broadly representative of populations who eat some fish, but not daily; nevertheless it is useful to compare our results with those from the Seychelles. Prior to this study, the Seychelles Child Development Study was the largest longitudinal study investigating the relationship between prenatal exposure to mercury and offspring behaviour through childhood and adolescence (Davidson et al., 1998, Davidson et al., 2011). This study was designed in a population of high mercury exposure (with daily fish consumption the norm), specifically to determine the adverse consequences of prenatal Hg exposure. Mercury exposure was estimated by analysis of maternal hair post-delivery as a proxy for prenatal exposure, and behaviour was determined at different ages from 5 to 17 years. At no stage did the authors find an adverse effect of prenatal mercury level on offspring behaviour – indeed the reverse was shown; the children and adolescents tended to have better behaviour with increasing prenatal exposure (Davidson et al., 2011), findings mirrored in the present study. Our study has differed from theirs in having about twice as many participants as well as being able to assess effects among the offspring of non-fish-eaters as well as fish eaters. The null associations in our study were found in offspring of non-fish-eaters as well as the fish eaters, thus weakening the possibility that nutrients in fish were negating any deterioration in behaviour that might be due to prenatal mercury levels.

### 4.1 Strengths and limitations

There are a number of strengths to this study: (i) the numbers studied are larger than in previous studies; (ii) the behaviour data were collected prospectively without any knowledge of exposure to mercury; (iii) information on behaviour was collected independently from the mothers and teachers, neither of whom knew the extent of prenatal mercury exposure; the SDQ measure used, though short, has been shown to be particularly accurate in regard to identifying clinically abnormal behaviours including hyperactivity; (iv) because the study was undertaken in a population with modest exposure levels we have been able to compare trends in offspring behaviour with levels of Hg exposure across the range from 0.24 to 12.76 μg/L, and children of women who ate fish with those who ate none; (v) maternal mercury levels were collected in the first half of pregnancy at a time when the developing brain is most susceptible to insults; (vi) unlike the studies of the Faroes and Seychelles, this study included a group of women who did not eat fish but nevertheless had blood mercury levels available for analysis; (vii) in order to ensure that the results relating to behaviour were not biased by maternal attitudes, we analysed the teacher’s independent assessment of the child; (viii) this is the only study with exposure relating to the first half of pregnancy, since others have used biological samples such as maternal hair or umbilical cord that are more likely to reflect third trimester exposures.

The limitations of this study include: (a) that the information on the frequency with which fish was consumed prenatally was obtained in the third trimester, whereas the blood mercury was measured on samples mainly collected in the first half of pregnancy. We think this is unlikely to cause a bias since it has been shown that maternal diets tend to be fairly stable over time (Northstone and Emmett, 2008). (b) The analysis of the prenatal blood for mercury took place 19 years after it was collected. Although it is conceivable that this would have biased the results, the levels achieved and the relationships with dietary and other factors such as dental mercury are similar to those found by others (Golding et al., 2013, Golding et al., 2016b). (c) Although we have allowed for a variety of factors associated with mercury levels and/or behaviour, it is possible that there are other confounders that should have been taken into account. However such unknown confounders would have to have major effects if they were to reveal an adverse association between mercury level and offspring behaviour. (d) We were limited to the types of behaviour measured in the SDQ. These will not reflect the behaviours shown in a randomised controlled experiment with pregnant primates who consumed methyl mercury in apple juice; the offspring showed an increased risk of non-social passive behaviour compared with controls whose mothers had been given apple juice with no added mercury; this difference in behaviour increased as the infant monkeys got older (Burbacher et al., 1990). If such an effect on non-social passive behaviour were to occur in humans, it might not be identified using the SDQ, or it might be revealed as a positive behaviour on the peer difficulties or hyperactive scales. (e) It remains possible that differences in metabolism, perhaps the result of different genotypes in either the mother or offspring, may have a confounding effect (e.g. Julvez et al., 2013) or make some individuals susceptible even when the population is not.

## 5 Conclusions

After assessing 126 possible associations between offspring behaviour and prenatal mercury exposure, and taking a P value of 0.10 to ensure no adverse effects were missed, only 18 were significant at this level after adjustment, and 16 of these indicated that the higher the mercury level, the more optimal the offspring’s behaviour. There were no detectable differences in the relationships of prenatal mercury and offspring behaviour between the children of women who ate fish in pregnancy and those who did not other than expected by chance. The inclusion of prenatal selenium exposure in the analyses made only marginal differences to the regression coefficients, implying that the results were not influenced by the strong association between mercury and blood selenium.

## Conflict of interest

None.

## Figures and Tables

**Table 1:** Mean [95% CI] unadjusted scores of offspring behaviour scales according to whether or not the mother ate fish prenatally (subjects with prenatal mercury measures).

Age of child and behaviour score	Ate no fish		Did eat fish		All	
	N	Mean [95% CI]	N	Mean [95% CI]	N	Mean [95% CI]
Total difficulties						
Age 47m^a^ (M)	356	9.23 [8.73, 9.72]	2285	8.39 [8.21, 8.57]	2776	8.55 [8.38, 8.72]
Age 81m^a^ (M)	299	8.47 [7.88, 9.06]	2036	7.12 [6.92, 7.32]	2436	7.32 [7.13, 7.51]
Age 7–8y^a^ (T)	223	6.55 [5.74, 7.36]	1287	5.25 [5.00, 5.55]	1692	5.64 [5.36, 5.91]
Age 10–11y^a^ (T)	261	6.63 [5.90, 7.36]	1478	5.39 [5.10, 5.69]	1959	5.72 [5.46, 5.98]
Age 11–12y^a^ (M)	244	7.46 [6.76, 8.16]	1730	6.12 [5.89, 6.34]	2062	6.36 [6.15, 6.57]
Age 13y^a^ (M)	222	7.49 [6.81, 8.17]	1635	6.36 [6.13, 6.59]	1942	6.49 [6.28, 6.71]
Age 16–17y (M)	173	6.24 [5.50, 6.98]	1369	5.95 [5.70, 6.21]	1599	6.01 [5.78, 6.25]

Prosocial						
Age 47m (M)	356	7.09 [6.88, 7.30]	2285	7.07 [6.99, 7.15]	2776	7.08 [7.01, 7.16]
Age 81m (M)	300	8.10 [7.90, 8.29]	2043	8.23 [8.16, 8.31]	2445	8.21 [8.14, 8.28]
Age 7–8y^a^ (T)	223	7.54 [7.19, 7.88]	1286	7.91 [7.78, 8.04]	1690	7.81 [7.69, 7.93]
Age 10–11y (T)	261	7.67 [7.37, 7.96]	1478	7.87 [7.75, 8.00]	1959	7.80 [7.69, 7.91]
Age 11–12y (M)	244	8.14 [7.92, 8.37]	1730	8.37 [8.29, 8.45]	2062	8.33 [8.26, 8.40]
Age 13y (M)	225	8.01 [7.75, 8.26]	1642	8.23 [8.15, 8.31]	1952	8.21 [8.13, 8.28]
Age 16–17y (M)	223	7.85 [7.55, 8.14]	1375	8.02 [7.92, 8.12]	1608	8.01 [7.92, 8.10]

Hyperactive						
Age 47m^a^ (M)	356	4.02 [3.78, 4.25]	2285	3.74 [3.64, 3.83]	2776	3.79 [3.70, 3.87]
Age 81m^a^ (M)	299	3.71 [3.41, 4.02]	2036	3.20 [3.10, 3.30]	2436	3.27 [3.18, 3.37]
Age 7–8y^a^ (T)	223	2.78 [2.40, 3.15]	1288	2.29 [2.15, 2.43]	1693	2.45 [2.32, 2.58]
Age 10–11y^a^ (T)	261	2.67 [2.32, 3.02]	1478	2.14 [2.01, 2.28]	1959	2.31 [2.19, 2.43]
Age 11–12y^a^ (M)	244	2.95 [2.66, 3.25]	1729	2.63 [2.52, 2.73]	2061	2.70 [2.60, 2.79]
Age 13y^a^ (M)	223	3.23 [2.93, 3.53]	1641	2.74 [2.64, 2.85]	1949	2.81 [2.71, 2.90]
Age 16–17y (M)	176	2.56 [2.23, 2.89]	1378	2.50 [2.39, 2.62]	1611	2.52 [2.42, 2.62]

Conduct						
Age 47m^a^ (M)	356	2.00 [1.86, 2.15]	2285	1.83 [1.77, 1.88]	2776	1.86 [1.81, 1.91]
Age 81m^a^ (M)	300	1.80 [1.62, 1.98]	2044	1.55 [1.49, 1.61]	2446	1.59 [1.53, 1.65]
Age 7–8y^a^ (T)	223	0.96 [0.73, 1.19]	1286	0.59 [0.52, 0.65]	1692	0.68 [0.62, 0.75]
Age 10–11y^a^ (T)	261	1.12 [0.89, 1.35]	1478	0.79 [0.71, 0.87]	1959	0.89 [0.81, 0.96]
Age 11–12y^a^ (M)	244	1.42 [1.23, 1.61]	1731	1.11 [1.05, 1.17]	2063	1.17 [1.11, 1.23]
Age 13y^a^ (M)	225	1.40 [1.21, 1.58]	1640	1.14 [1.08, 1.21]	1950	1.18 [1.12, 1.23]
Age 16–17 (M)	178	0.97 [0.78, 1.15]	1376	0.94 [0.87, 1.01]	1611	0.95 [0.89, 1.01]

Emotional						
Age 47 m (M)	356	1.43 [1.28, 1.58]	2285	1.37 [1.31, 1.43]	2776	1.40 [1.34, 1.45]
Age 81 m (M)	300	1.65 [1.46, 1.84]	2042	1.47 [1.40, 1.54]	2444	1.50 [1.44, 1.56]
Age 7–8y^a^ (T)	223	1.56 [1.28, 1.84]	1288	1.26 [1.16, 1.36]	1693	1.33 [1.24, 1.42]
Age 10–11y^a^ (T)	261	1.48 [1.24, 1.72]	1477	0.79 [0.71, 0.87]	1958	1.29 [1.20, 1.37]
Age 11–12y (M)	242	1.59 [1.35, 1.82]	1727	1.39 [1.31, 1.46]	2057	1.42 [1.35, 1.50]
Age 13y (M)	227	1.36 [1.13, 1.59]	1640	1.37 [1.28, 1.45]	1952	1.37 [1.29, 1.45]
Age 16–17y (M)	176	1.58 [1.29, 1.87]	1375	1.47 [1.37, 1.57]	1608	1.49 [1.39, 1.58]

Peer problems						
Age 47m^a^ (M)	356	1.78 [1.61, 1.95]	2285	1.45 [1.39, 1.51]	2776	1.51 [1.46, 1.57]
Age 81m^a^ (M)	299	1.42 [1.24, 1.60]	2043	0.98 [0.92, 1.04]	2444	1.05 [0.99, 1.11]
Age 7–8y (T)	223	1.28 [1.05, 1.51]	1288	1.13 [1.04, 1.23]	1693	1.19 [1.10, 1.27]
Age 10–11y (T)	261	1.37 [1.16, 1.59]	1478	1.21 [1.12, 1.31]	1959	1.24 [1.16, 1.32]
Age 11–12y^a^ (M)	244	1.51 [1.27, 1.75]	1733	1.02 [0.95, 1.09]	2065	1.09 [1.03, 1.16]
Age 13y^a^ (M)	226	1.60 [1.35, 1.85]	1641	1.14 [1.06, 1.21]	1952	1.18 [1.11, 1.25]
Age 16–17y (M)	176	1.32 [1.04, 1.59]	1375	1.09 [1.01, 1.17]	1608	1.11 [1.04, 1.19]

M = Mother; T = Teacher assessment.

For all scales except the prosocial behaviour, the higher the score, the worse the behaviour.

a Differences between columns 1 and 2: P < 0.05.

**Table 2:** Relationship between prenatal maternal blood mercury and offspring scores on Difficult Behaviour SDQ scale at various ages; positive βs indicate increasingly poor behaviour with increasing maternal blood mercury. Highlighted are results with P < 0.100.

Age of Child and Prenatal Fish Eating	Unadjusted			Adjusted model A			Adjusted model B		
	N	β [95% CI]	P	N	β [95% CI	P	N	β [95% CI]	P
Age 47m (M)									
Non-fish eaters	356	−0.92 [−1.58,−0.27]	0.006	298	−0.61 [−1.31,+0.10]	0.090	298	−0.60 [−1.31,+0.11]	0.097
Fish eaters	2285	−0.39 [−0.55,−0.22]	<0.001	2025	−0.26 [−0.44,−0.08]	0.004	2025	−0.26 [−0.44,−0.07]	0.006
All	2776	−0.49 [−0.65,−0.34]	<0.001	2331	−0.29 [−0.46,−0.12]	0.001	2331	−0.29 [−0.46,−0.12]	0.001

Age 81m (M)									
Non-fish eaters	299	+0.18 [−0.59,+0.95]	0.654	256	+0.19 [−0.67,+1.05]	0.658	256	+0.19 [−0.68,+1.05]	0.673
Fish eaters	2036	−0.18 [−0.36,+0.00]	0.054	1817	−0.05 [−0.24,+0.15]	0.645	1817	−0.06 [−0.26,+0.14]	0.534
All	2436	−0.26 [−0.43,−0.09]	0.003	2080	−0.10 [−0.29,+0.09]	0.293	2080	−0.12 [−0.31,+0.08]	0.235

Age 7–8y (T)									
Non-fish eaters	223	+0.40 [−0.41,+1.22]	0.331	181	+0.36 [−0.96,+1.68]	0.590	181	+0.36 [−0.97,+1.69]	0.593
Fish eaters	1287	−0.25 [−0.53,+0.03]	0.080	1108	−0.11 [−0.41,+0.19]	0.487	1108	−0.11 [−0.42,+0.21]	0.514
All	1692	−0.38 [−0.63,−0.13]	0.003	1297	−0.13 [−0.42,+0.15]	0.358	1297	−0.13 [−0.42,+0.17]	0.401
				
Age 10–11y (T)									
Non-fish eaters	261	+0.39 [−0.27,+1.06]	0.242	202	+0.43 [−0.55,+1.41]	0.392	202	+0.43 [−0.56,+1.41]	0.392
Fish eaters	1478	−0.38 [−0.65,−0.10]	0.007	1265	+0.01 [−0.28,+0.29]	0.961	1265	+0.04 [−0.25,+0.34]	0.766
All	1959	−0.41 [−0.64,−0.18]	0.001	1476	+0.04 [−0.22,+0.30]	0.757	1476	+0.08 [−0.20,+0.35]	0.589

Age 11–12y (M)									
Non-fish eaters	244	−0.34 [−1.26,+0.58]	0.471	209	−0.02 [−0.98,+0.94]	0.962	209	+0.01 [−0.95,+0.98]	0.977
Fish eaters	1730	−0.15 [−0.36,+0.05]	0.146	1580	+0.03 [−0.18,+0.25]	0.755	1580	+0.03 [−0.20,+0.24]	0.826
All	2062	−0.27 [−0.46,−0.08]	0.005	1796	−0.03 [−0.23,+0.18]	0.798	1796	−0.03 [−0.24,+0.19]	0.820

Age 13y (M)									
Non-fish eaters	222	−0.42 [−1.27,+0.43]	0.331	190	−0.29 [−1.23, +0.65]	0.547	190	−0.18 [−1.12,+0.77]	0.712
Fish eaters	1635	−0.22 [−0.43,−0.01]	0.044	1485	−0.04 [−0.26,+0.18]	0.728	1485	−0.03 [−0.26,+0.19]	0.776
All	1942	−0.30 [−0.48,−0.11]	0.002	1682	−0.10 [−0.31,+0.11]	0.336	1682	−0.07 [−0.29,+0.15]	0.525

Age 16–17y (M)									
Non-fish eaters	173	−0.41 [−1.40,+0.57]	0.410	150	−0.23 [−1.36,+0.89]	0.682	150	−0.22 [−1.35,+0.91]	0.704
Fish eaters	1369	−0.16 [−0.40,+0.07]	0.178	1252	−0.08 [−0.32,+0.16]	0.515	1252	−0.05 [−0.30,+0.21]	0.723
All	1599	−0.21 [−0.42,+0.01]	0.061	1407	−0.09 [−0.32,+0.14]	0.442	1407	−0.05 [−0.29,+0.19]	0.689

M = Mother; T = Teacher assessment.

β indicates the change in units of offspring behaviour score as the prenatal blood mercury increases by 1SD. A positive score indicates that the behaviour deteriorates as the mother’s blood mercury increased.

Model A = Adjustment for family adversity, housing tenure, overcrowding, stressful life events, maternal smoking, alcohol consumption, maternal age, parity, maternal education, breast feeding and sex.

Model B = Model A + adjustment for maternal prenatal blood selenium level.

**Table 3:** Summary of all results of adjusted* associations where at least one of the three groups (mother ate fish, mother did not eat fish, all mothers) was significant at the 0.10 level (from Supplementary Tables 1–5). Highlighted are results with P < 0.100.

Age of child and behaviour score	Ate no fish			Did eat fish			All		
	N	β [95% CI]	P	N	β [95% CI]	P	N	β Mean [95% CI]	P
47m Hyperactive (M)	298	−0.23 [−0.58,+0.12]	0.195	2025	−0.08 [−0.18,+0.01]	0.085	2331	−0.08 [−0.17,+0.01]	0.074
47m Conduct (M)	298	+0.10 [−0.11,+0.31]	0.366	2025	−0.08 [−0.13,−0.02]	0.007	2331	−0.06 [−0.11,−0.01]	0.024
10–11y Conduct (T)	202	+0.25 [−0.04,+0.53]	0.089	1265	+0.03 [−0.05,+0.10]	0.524	1476	+0.05 [−0.02,+0.12]	0.167
47m Emotional (M)	298	−0.20 [−0.43,+0.03]	0.095	2025	+0.05 [−0.12,+0.01]	0.113	2331	−0.06 [−0.12,−0.00]	0.048
47m Peer Problems (M)	298	−0.27 [−0.52,−0.01]	0.040	2025	−0.06 [−0.12, +0.00]	0.059	2331	−0.08 [−0.14,−0.02]	0.006
81m Peer Problems	256	−0.04 [−0.31,+0.23]	0.774	1821	−0.08 [−0.14,−0.02]	0.010	2084	−0.10 [−0.16,−0.04]	0.001
(M)									
13y Peer Problems	194	−0.22 [−0.57,+0.13]	0.209	1490	−0.07 [−0.14,+0.00]	0.063	1691	−0.08 [−0.16,−0.01]	0.027
(M)									
10–11y Prosocial	202	−0.33 [−0.71,+0.05]	0.084	1265	−0.03 [−0.16,+0.10]	0.624	1476	−0.06 [−0.18,+0.06]	0.315
(T)									

M = mother completed; T = teacher completed.

β indicates the change in units of offspring behaviour score as the prenatal blood mercury increases by 1SD. A positive score indicates that the behaviour deteriorates as the mother’s prenatal blood mercury increased for all behaviour scores except the prosocial score.

*Adjustment for family adversity, housing tenure, overcrowding, stressful life events, maternal smoking, alcohol consumption, maternal age, parity, maternal education, maternal prenatal blood selenium level, breast feeding and sex.
