# Supplementary material for: Prenatal mercury exposure and offspring behaviour in childhood and adolescence
Source: Neurotoxicology. 2016 Dec;57:87–94. doi: 10.1016/j.neuro.2016.09.003 (PMC5138154; doi:10.1016/j.neuro.2016.09.003)
Supplement: Supplementary file 1 [file mmc1.docx]

Supplementary Table 1: Relationship between prenatal maternal blood mercury and offspring scores on **hyperactive** behaviour SDQ scale.

Highlighted are results with P<0.100.

| **Age of Child and Prenatal** | **UNADJUSTED** | | | **ADJUSTED*** | | |
| --- | --- | --- | --- | --- | --- | --- |
| **Fish Eating** | **N** | **β [95% CI]** | **P** | **N** | **β [95% CI]** | **P** |
|  |  |  |  |  |  |  |
| **Age 47m (M)** |  |  |  |  |  |  |
| Non-fish eaters | 356 | **-0.35 [-0.67,-0.04]** | **0.030** | 298 | -0.23 [-0.58,+0.12] | 0.195 |
| Fish eaters | 2285 | **-0.17 [-0.26,-0.08]** | **<0.001** | 2025 | **-0.08 [-0.18,+0.01]** | **0.085** |
| All | 2776 | **-0.19 [-0.27,-0.11]** | **<0.001** | 2331 | **-0.08 [-0.17,+0.01]** | **0.074** |
|  |  |  |  |  |  |  |
| **Age 81m (M)** |  |  |  |  |  |  |
| Non-fish eaters | 299 | +0.21 [-0.20,+0.61] | 0.314 | 256 | +0.19 [-0.25,+0.62] | 0.401 |
| Fish eaters | 2036 | **-0.09 [-0.18,+0.00]** | **0.053** | 1817 | -0.00 [-0.10,+0.10] | 0.964 |
| All | 2436 | **-0.11 [-0.19,-0.02]** | **0.014** | 2080 | -0.14 [-0.11,+0.08] | 0.773 |
|  |  |  |  |  |  |  |
| **Age 7-8y (T)** |  |  |  |  |  |  |
| Non-fish eaters | 223 | +0.19 [-0.19,+0.58] | 0.315 | 181 | +0.48 [-0.10,+1.06] | 0.105 |
| Fish eaters | 1288 | **-0.13 [-0.26,-0.00]** | **0.050** | 1108 | -0.09 [-0.23,+0.06] | 0.235 |
| All | 1693 | **-0.17 [-0.29,-0.06]** | **<0.001** | 1297 | -0.05 [-0.18,+0.09] | 0.496 |
|  |  |  |  |  |  |  |
| **Age 10-11y (T)** |  |  |  |  |  |  |
| Non-fish eaters | 261 | +0.16 [-0.15,+0.48] | 0.311 | 202 | +0.15 [-0.30,+0.59] | 0.523 |
| Fish eaters | 1478 | **-0.13 [-0.25,-0.00]** | **0.044** | 1265 | +0.09 [-0.04,+0.22] | 0.188 |
| All | 1959 | **-0.14 [-0.25,-0.04]** | **0.008** | 1476 | +0.09 [-0.03,+0.21] | 0.161 |
|  |  |  |  |  |  |  |
| **Age 11-12y (M)** |  |  |  |  |  |  |
| Non-fish eaters | 244 | -0.06 [-0.45,+0.33] | 0.752 | 209 | +0.06 [-0.35,+0.46] | 0.791 |
| Fish eaters | 1729 | -0.07 [-0.16,+0.02] | 0.144 | 1580 | +0.01 [-0.09,+0.11] | 0.832 |
| All | 2061 | **-0.10 [-0.19,-0.01]** | **0.022** | 1796 | +0.01 [-0.09,+0.10] | 0.879 |
|  |  |  |  |  |  |  |
|  |  |  |  |  |  |  |
|  |  |  |  |  |  |  |
|  |  |  |  |  |  |  |
| **Age 13y (M)** |  |  |  |  |  |  |
| Non-fish eaters | 223 | +0.02 [-0.35,+0.39] | 0.910 | 191 | +0.16 [-0.26,+0.57] | 0.458 |
| Fish eaters | 1641 | -0.02 [-0.11,+0.08] | 0.720 | 1491 | +0.06 [-0.05,+0.16] | 0.286 |
| All | 1949 | -0.05 [-0.13,+0.04] | 0.271 | 1689 | +0.05 [-0.05,+0.14] | 0.355 |
|  |  |  |  |  |  |  |
| **Age 16-17y (M)** |  |  |  |  |  |  |
| Non-fish eaters | 176 | -0.06 [-0.50,+0.38] | 0.797 | 153 | +0.12 [-0.35,+0.59] | 0.605 |
| Fish eaters | **1378** | **-0.09 [-0.20,+0.01]** | **0.082** | 1261 | -0.05 [-0.17,+0.06] | 0.383 |
| All | **1611** | **-0.10 [-0.19,-0.00]** | **0.048** | 1419 | -0.02 [-0.13,+0.09] | 0.662 |
|  |  |  |  |  |  |  |

M = Mother; T = Teacher

β indicates the change in units of offspring behaviour score as the prenatal blood mercury increases by 1SD. A positive score indicates that the behaviour deteriorates as the mother’s blood mercury increased.

*Adjustment for family adversity, housing tenure, overcrowding, stressful life events, maternal smoking, alcohol consumption, maternal age, parity, maternal education, maternal prenatal blood selenium level, breast feeding and sex.

Supplementary Table 2: Relationship between prenatal maternal blood mercury and offspring scores on **conduct problems** SDQ scale. Highlighted are results with P<0.100.

| **Age of Child and Prenatal** | **UNADJUSTED** | | | **ADJUSTED*** | | |
| --- | --- | --- | --- | --- | --- | --- |
| **Fish Eating** | **N** | **β [95% CI]** | **P** | **N** | **β [95% CI]** | **P** |
|  |  |  |  |  |  |  |
| **Age 47m (M)** |  |  |  |  |  |  |
| Non-fish eaters | 356 | -0.09 [-0.28,+0.11] | 0.378 | 298 | +0.10 [-0.11,+0.31] | 0.366 |
| Fish eaters | 2285 | **-0.10 [-0.15,-0.05]** | **<0.001** | 2025 | **-0.08 [-0.13,-0.02]** | **0.007** |
| All | 2776 | **-0.10 [-0.15,-0.06]** | **<0.001** | 2331 | **-0.06 [-0.11,-0.01]** | **0.024** |
|  |  |  |  |  |  |  |
| **Age 81m (M)** |  |  |  |  |  |  |
| Non-fish eaters | 300 | +0.16 [-0.08,+0.40] | 0.185 | 257 | +0.20 [-0.06,+0.46] | 0.134 |
| Fish eaters | 2044 | -0.03 [-0.09,+0.03] | 0.267 | 1822 | +0.02 [-0.05,+0.08] | 0.633 |
| All | 2446 | -0.04 [-0.09,+0.01] | 0.154 | 2086 | +0.02 [-0.04,+0.08] | 0.502 |
|  |  |  |  |  |  |  |
| **Age 7-8y (T)** |  |  |  |  |  |  |
| Non-fish eaters | 222 | +0.05 [-0.19,+0.29] | 0.665 | 181 | +0.22 [-0.16,+0.60] | 0.258 |
| Fish eaters | 1286 | **-0.09 [-0.16,-0.03]** | **0.004** | 1108 | -0.06 [-0.13,+0.02] | 0.130 |
| All | 1692 | **-0.12 [-0.18,-0.05]** | **<0.001** | 1297 | -0.06 [-0.13,+0.02] | 0.126 |
|  |  |  |  |  |  |  |
| **Age 10-11y (T)** |  |  |  |  |  |  |
| Non-fish eaters | 261 | **+0.18 [-0.03,+0.39]** | **0.095** | **202** | **+0.25 [-0.04,+0.53]** | **0.089** |
| Fish eaters | 1478 | **-0.09 [-0.17,-0.02]** | **0.015** | 1265 | +0.03 [-0.05,+0.10] | 0.524 |
| All | 1959 | **-0.09 [-0.16,-0.03]** | **0.005** | 1476 | +0.05 [-0.02,+0.12] | 0.167 |
|  |  |  |  |  |  |  |
| **Age 11-12y (M)** |  |  |  |  |  |  |
| Non-fish eaters | 244 | +0.04 [-0.21,+0.29] | 0.757 | 209 | +0.18 [-0.09,+0.45] | 0.183 |
| Fish eaters | 1731 | -0.01 [-0.06,+0.05] | 0.814 | 1580 | +0.05 [-0.02,+0.11] | 0.138 |
| All | 2063 | -0.03 [-0.08,+0.03] | 0.327 | 1796 | +0.04 [-0.02,+0.10] | 0.165 |
|  |  |  |  |  |  |  |
|  |  |  |  |  |  |  |
|  |  |  |  |  |  |  |
|  |  |  |  |  |  |  |
| **Age 13y (M)** |  |  |  |  |  |  |
| Non-fish eaters | 225 | -0.10 [-0.34,+0.13] | 0.385 | 193 | -0.02 [-0.27,+0.24] | 0.911 |
| Fish eaters | 1640 | -0.04 [-0.10,+0.02] | 0.143 | 1489 | +0.01 [-0.06,+0.07] | 0.813 |
| All | **1950** | **-0.06 [-0.11,-0.01]** | **0.026** | 1689 | +0.00 [-0.06,+0.06] | 0.968 |
|  |  |  |  |  |  |  |
| **Age 16-17y (M)** |  |  |  |  |  |  |
| Non-fish eaters | 178 | -0.12 [-0.37,+0.13] | 0.353 | 155 | -0.03 [-0.32,+0.25] | 0.814 |
| Fish eaters | 1376 | +0.02 [-0.04,+0.08] | 0.523 | 1259 | +0.03 [-0.04,+0.10] | 0.347 |
| All | 1611 | +0.01 [-0.05,+0.07] | 0.703 | 1419 | +0.03 [-0.03,+0.10] | 0.299 |

M = Mother; T = Teacher

β indicates the change in units of offspring behaviour score as the prenatal blood mercury increases by 1SD. A positive score indicates that the behaviour deteriorates as the mother’s blood mercury increased.

*Adjustment for family adversity, housing tenure, overcrowding, stressful life events, maternal smoking, alcohol consumption, maternal age, parity, maternal education, maternal prenatal blood selenium level, breast feeding and sex.

Supplementary Table 3: Relationship between prenatal maternal blood mercury and offspring scores on **emotional symptoms** SDQ scale. Highlighted are results with P<0.100.

| **Age of Child and Prenatal** | **UNADJUSTED** | | | **ADJUSTED*** | | |
| --- | --- | --- | --- | --- | --- | --- |
| **Fish Eating** | **N** | **β [95% CI]** | **P** | **N** | **β [95% CI]** | **P** |
|  |  |  |  |  |  |  |
| **Age 47m (M)** |  |  |  |  |  |  |
| Non-fish eaters | 356 | **-0.22 [-0.42,-0.02]** | **0.035** | 298 | **-0.20 [-0.43,+0.03]** | **0.095** |
| Fish eaters | 2285 | -0.02 [-0.08,+0.03] | 0.435 | 2025 | +0.05 [-0.12,+0.01] | 0.113 |
| All | 2776 | **-0.06 [-0.11,-0.00]** | **0.035** | 2331 | **-0.06 [-0.12,-0.00]** | **0.048** |
|  |  |  |  |  |  |  |
| **Age 81m (M)** |  |  |  |  |  |  |
| Non-fish eaters | 300 | -0.08 [-0.33,+0.17] | 0.537 | 257 | -0.14 [-0.42,+0.14] | 0.336 |
| Fish eaters | 2042 | +0.02 [-0.04,+0.09] | 0.524 | 1822 | +0.02 [-0.06,+0.09] | 0.663 |
| All | 2444 | -0.02 [-0.08,+0.04] | 0.615 | 2086 | -0.01 [-0.08,+0.06] | 0.767 |
|  |  |  |  |  |  |  |
| **Age 7-8y (T)** |  |  |  |  |  |  |
| Non-fish eaters | 223 | -0.10 [-0.38,+0.19] | 0.495 | 181 | -0.33 [-0.83,+0.18] | 0.202 |
| Fish eaters | 1288 | +0.02 [-0.07,+0.11] | 0.696 | 1108 | +0.06 [-0.05,+0.16] | 0.297 |
| All | 1693 | -0.05 [-0.14,+0.03] | 0.187 | 1297 | -0.00 [-0.11,+0.10] | 0.952 |
|  |  |  |  |  |  |  |
| **Age 10-11y (T)** |  |  |  |  |  |  |
| Non-fish eaters | 261 | -0.09 [-0.31,+0.14] | 0.449 | 202 | -0.14 [-0.47,+0.20] | 0.421 |
| Fish eaters | 1477 | **-0.09 [-0.18,-0.00]** | **0.049** | 1265 | -0.04 [-0.14,+0.06] | 0.433 |
| All | 1958 | **-0.12 [-0.19,-0.04]** | **0.002** | 1476 | -0.04 [-0.14,+0.05] | 0.380 |
|  |  |  |  |  |  |  |
| **Age 11-12y (M)** |  |  |  |  |  |  |
| Non-fish eaters | 242 | -0.11 [-0.42,+0.20] | 0.474 | 208 | -0.07 [-0.41,+0.28] | 0.694 |
| Fish eaters | 1727 | -0.03 [-0.10,+0.04] | 0.424 | 1577 | +0.01 [-0.07,+0.08] | 0.877 |
| All | 2057 | **-0.06 [-0.13,+0.01]** | **0.075** | 1792 | -0.02 [-0.09,+0.06] | 0.704 |
|  |  |  |  |  |  |  |
|  |  |  |  |  |  |  |
|  |  |  |  |  |  |  |
|  |  |  |  |  |  |  |
| **Age 13y (M)** |  |  |  |  |  |  |
| Non-fish eaters | 227 | +0.01 [-0.28,+0.29] | 0.959 | 194 | -0.05 [-0.36,+0.25] | 0.725 |
| Fish eaters | 1640 | -0.05 [-0.13,+0.02] | 0.163 | 1490 | -0.03 [-0.11,+0.06] | 0.509 |
| All | **1952** | **-0.06 [-0.13,+0.01]** | **0.089** | 1691 | -0.02 [-0.10,+0.06] | 0.545 |
|  |  |  |  |  |  |  |
| **Age 16-17y (M)** |  |  |  |  |  |  |
| Non-fish eaters | 176 | -0.18 [-0.56,+0.21] | 0.369 | 153 | -0.24 [-0.66,+0.18] | 0.264 |
| Fish eaters | 1375 | -0.01 [-0.11,+0.08] | 0.795 | 1258 | +0.03 [-0.08,+0.13] | 0.610 |
| All | 1608 | -0.04 [-0.12,+0.05] | 0.378 | 1416 | +0.01 [-0.09,+0.10] | 0.923 |
|  |  |  |  |  |  |  |

M = Mother; T = Teacher

β indicates the change in units of offspring behaviour score as the prenatal blood mercury increases by 1SD. A positive score indicates that the behaviour deteriorates as the mother’s blood mercury increased.

*Adjustment for family adversity, housing tenure, overcrowding, stressful life events, maternal smoking, alcohol consumption, maternal age, parity, maternal education, maternal prenatal blood selenium level, breast feeding and sex.

Supplementary Table 4: Relationship between prenatal maternal blood mercury and offspring scores on **Peer Problems** SDQ scale. Highlighted are results with P<0.100.

| **Age of Child and Prenatal** | **UNADJUSTED** | | | **ADJUSTED*** | | |
| --- | --- | --- | --- | --- | --- | --- |
| **Fish Eating** | **N** | **β [95% CI]** | **P** | **N** | **β [95% CI]** | **P** |
|  |  |  |  |  |  |  |
| **Age 47m (M)** |  |  |  |  |  |  |
| Non-fish eaters | 356 | **-0.27 [-0.50,-0.04]** | **0.021** | 298 | **-0.27 [-0.52,-0.01]** | **0.040** |
| Fish eaters | 2285 | **-0.10 [-0.15,-0.04]** | **0.001** | 2025 | **-0.06 [-0.12,+0.00]** | **0.059** |
| All | 2776 | **-0.14 [-0.19,-0.09]** | **<0.001** | 2331 | **-0.08 [-0.14,-0.02]** | **0.006** |
|  |  |  |  |  |  |  |
| **Age 81m (M)** |  |  |  |  |  |  |
| Non-fish eaters | 299 | -0.06 [-0.29,+0.18] | 0.637 | 256 | -0.04 [-0.31,+0.23] | 0.774 |
| Fish eaters | 2043 | **-0.08 [-0.13,-0.02]** | **0.005** | 1821 | **-0.08 [-0.14,-0.02]** | **0.010** |
| All | 2444 | **-0.10 [-0.15,-0.05]** | **<0.001** | 2084 | **-0.10 [-0.16,-0.04]** | **0.001** |
|  |  |  |  |  |  |  |
| **Age 7-8y (T)** |  |  |  |  |  |  |
| Non-fish eaters | 223 | **+0.25 [+0.03,+0.48]** | **0.029** | 181 | -0.01 [-0.42,+0.39] | 0.949 |
| Fish eaters | 1288 | -0.04 [-0.14,+0.05] | 0.379 | 1108 | -0.02 [-0.13,+0.09] | 0.738 |
| All | 1693 | -0.04 [-0.12,+0.04] | 0.361 | 1297 | -0.02 [-0.12,+0.08] | 0.673 |
|  |  |  |  |  |  |  |
| **Age 10-11y (T)** |  |  |  |  |  |  |
| Non-fish eaters | 261 | +0.13 [-0.06,+0.33] | 0.180 | 202 | +0.17 [-0.13,+0.46] | 0.277 |
| Fish eaters | 1478 | -0.07 [-0.16,+0.02] | 0.119 | 1265 | -0.04 [-0.14,+0.06] | 0.440 |
| All | 1959 | -0.06 [-0.13,+0.02] | 0.125 | 1476 | -0.02 [-0.12,+0.07] | 0.638 |
|  |  |  |  |  |  |  |
| **Age 11-12y (M)** |  |  |  |  |  |  |
| Non-fish eaters | 244 | -0.20 [-0.52,+0.12] | 0.214 | 209 | -0.14 [-0.47,+0.18] | 0.378 |
| Fish eaters | 1733 | -0.05 [-0.11,+0.02] | 0.137 | 1581 | -0.03 [-0.10,+0.04] | 0.354 |
| All | 2062 | **-0.09 [-0.15,-0.03]** | **0.006** | 1797 | -0.05 [-0.12,+0.02] | 0.130 |
|  |  |  |  |  |  |  |
|  |  |  |  |  |  |  |
|  |  |  |  |  |  |  |
|  |  |  |  |  |  |  |
| **Age 13y (M)** |  |  |  |  |  |  |
| Non-fish eaters | **226** | **-0.28 [-0.59,+0.04]** | **0.086** | 194 | -0.22 [-0.57,+0.13] | 0.209 |
| Fish eaters | **1641** | **-0.10 [-0.17,-0.03]** | **0.005** | **1490** | **-0.07 [-0.14, +0.00]** | **0.063** |
| All | **1952** | **-0.13 [-0.19,-0.06]** | **<0.001** | **1691** | **-0.08 [-0.16,-0.01]** | **0.027** |
|  |  |  |  |  |  |  |
| **Age 16-17y (M)** |  |  |  |  |  |  |
| Non-fish eaters | 176 | -0.20 [-0.57,+0.17] | 0.290 | 153 | -0.16 [-0.58,+0.26] | 0.456 |
| Fish eaters | **1375** | **-0.06 [-0.14,+0.01]** | **0.087** | 1258 | -0.06 [-0.14,+0.02] | 0.143 |
| All | **1608** | **-0.09 [-0.16,-0.02]** | **0.012** | 1416 | -0.06 [-0.15,+0.02] | 0.123 |
|  |  |  |  |  |  |  |

M = Mother; T = Teacher

β indicates the change in units of offspring behaviour score as the prenatal blood mercury increases by 1SD. A positive score indicates that the behaviour deteriorates as the mother’s blood mercury increased.

*Adjustment for family adversity, housing tenure, overcrowding, stressful life events, maternal smoking, alcohol consumption, maternal age, parity, maternal education, maternal prenatal blood selenium level, breast feeding and sex.

Supplementary Table 5: Relationship between prenatal maternal blood mercury and offspring scores on **prosocial behaviour** SDQ scale. Highlighted are results with P<0.100.

| **Age of Child and Prenatal** | **UNADJUSTED** | | | **ADJUSTED*** | | |
| --- | --- | --- | --- | --- | --- | --- |
| **Fish Eating** | **N** | **β [95% CI]** | **P** | **N** | **β [95% CI]** | **P** |
|  |  |  |  |  |  |  |
| **Age 47m (M)** |  |  |  |  |  |  |
| Non-fish eaters | 356 | +0.22 [-0.06,+0.50] | 0.129 | 298 | +0.19 [-0.12,+0.50] | 0.229 |
| Fish eaters | 2285 | +0.04 [-0.04,+0.12] | 0.319 | 2025 | +0.04 [-0.05,+0.13] | 0.377 |
| All | 2776 | +0.05 [-0.02,+0.12] | 0.130 | 2331 | +0.04 [-0.04,+0.12] | 0.322 |
|  |  |  |  |  |  |  |
| **Age 81m (M)** |  |  |  |  |  |  |
| Non-fish eaters | 300 | -0.15 [-0.41,+0.10] | 0.233 | 257 | -0.04 [-0.33,+0.24] | 0.772 |
| Fish eaters | 2043 | -0.04 [-0.11,+0.03] | 0.304 | 1821 | +0.01 [-0.07,+0.08] | 0.885 |
| All | 2445 | -0.02 [-0.09,+0.04] | 0.463 | 2085 | +0.00 [-0.07,+0.07] | 0.997 |
|  |  |  |  |  |  |  |
| **Age 7-8y (T)** |  |  |  |  |  |  |
| Non-fish eaters | 223 | **-0.33 [-0.68,+0.02]** | **0.064** | 181 | -0.15 [-0.72,+0.42] | 0.609 |
| Fish eaters | 1286 | -0.03 [-0.15,+0.09] | 0.626 | 1108 | -0.04 [-0.17,+0.10] | 0.613 |
| All | 1690 | +0.01 [-0.09,+0.12] | 0.789 | 1297 | -0.05 [-0.18,+0.08] | 0.411 |
|  |  |  |  |  |  |  |
| **Age 10-11y (T)** |  |  |  |  |  |  |
| Non-fish eaters | 261 | **-0.34 [-0.60,-0.08]** | **0.012** | 202 | **-0.33 [-0.71,+0.05]** | **0.084** |
| Fish eaters | 1478 | +0.02 [-0.09,+0.14] | 0.689 | 1265 | -0.03 [-0.16,+0.10] | 0.624 |
| All | 1959 | -0.01 [-0.10,+0.09] | 0.898 | 1476 | -0.06 [-0.18,+0.06] | 0.315 |
|  |  |  |  |  |  |  |
| **Age 11-12y (M)** |  |  |  |  |  |  |
| Non-fish eaters | 244 | +0.01 [-0.30,+0.30] | 0.972 | 209 | -0.08 [-0.41,+0.24] | 0.616 |
| Fish eaters | 1730 | -0.03 [-0.10,+0.04] | 0.339 | 1581 | -0.01 [-0.09,+0.07] | 0.824 |
| All | 2062 | -0.02 [-0.08,+0.05] | 0.619 | 1797 | -0.00 [-0.08,+0.07] | 0.921 |
|  |  |  |  |  |  |  |
|  |  |  |  |  |  |  |
|  |  |  |  |  |  |  |
|  |  |  |  |  |  |  |
| **Age 13y (M)** |  |  |  |  |  |  |
| Non-fish eaters | 225 | +0.17 [-0.15,+0.49] | 0.288 | 193 | +0.18 [-0.18,+0.54] | 0.332 |
| Fish eaters | 1642 | -0.05 [-0.12,+0.03] | 0.233 | 1491 | -0.01 [-0.09,+0.07] | 0.802 |
| All | 1952 | -0.02 [-0.09,+0.05] | 0.561 | 1691 | +0.01 [-0.07,+0.09] | 0.860 |
|  |  |  |  |  |  |  |
| **Age 16-17y (M)** |  |  |  |  |  |  |
| Non-fish eaters | 176 | +0.28 [-0.12,+0.67] | 0.173 | 154 | +0.28 [-0.17,+0.72] | 0.216 |
| Fish eaters | 1375 | +0.05 [-0.04,+0.14] | 0.296 | 1258 | +0.06 [-0.05,+0.16] | 0.298 |
| All | 1608 | +0.07 [-0.02,+0.15] | 0.122 | 1417 | +0.06 [-0.03,+0.16] | 0.198 |

M = Mother; T = Teacher

β indicates the change in units of offspring prosocial behaviour score as the prenatal blood mercury increases by 1SD. A positive score indicates that the behaviour improved as the mother’s blood mercury increased.

*Adjustment for family adversity, housing tenure, overcrowding, stressful life events, maternal smoking, alcohol consumption, maternal age, parity, maternal education, maternal prenatal blood selenium level, breast feeding and sex.
